# Early life socioeconomic environment and mammographic breast density

**DOI:** 10.1186/s12885-016-3010-x

**Published:** 2017-01-10

**Authors:** Parisa Tehranifar, Barbara A. Cohn, Julie D. Flom, Angeline Protacio, Piera Cirillo, L. H. Lumey, Karin B. Michels, Mary Beth Terry

**Affiliations:** 1 Department of Epidemiology, Columbia University Mailman School of Public Health, 722 West 168th St, New York, NY 10032 USA; 2 Herbert Irving Comprehensive Cancer Center, Columbia University Medical Center, New York, NY USA; 3 The Center for Research on Women and Children’s Health, The Child Health and Development Studies, Public Health Institute, Berkeley, CA USA; 4 The Imprints Center for Genetic and Environmental Lifecourse Studies, Columbia University Mailman School of Public Health, New York, NY USA; 5 Department of Epidemiology, University of California (UCLA) Fielding School of Public Health, Los Angeles, CA USA; 6 Institute for Prevention and Cancer Epidemiology, Freiburg University Medical Center, Freiburg, Germany

**Keywords:** Life-course, Socioeconomic status, Mammographic breast density, Reproductive factors, Lifestyles

## Abstract

**Background:**

Early life social environment may influence breast cancer through shaping risk factors operating in early life, adolescence and adulthood, or may be associated with breast cancer risk independent of known risk factors. We investigated the associations between early life socioeconomic status (SES) and mammographic density, a strong risk factor for breast cancer, and the extent to which these associations were independent of risk factors across the lifecourse.

**Methods:**

We used data from an adult follow-up study of two U.S. birth cohorts of women (average age = 43 years) with prospectively collected data starting during the pregnancy of the mother and continuing through early childhood of the offspring. We collected data on factors in later life periods through computer-assisted interviews with the offspring as adults, and obtained routine clinical mammograms for measurement of percent density and dense and nondense breast areas using a computer assisted method. We used generalized estimating equation models for multivariable analysis to account for correlated data for sibling sets within the study sample (*n* = 700 composed of 441 individuals and 127 sibling sets).

**Results:**

Highest vs. lowest family income level around the time of birth was associated with smaller dense breast area after adjustment for early life factors (e.g., birthweight, maternal smoking during pregnancy) and risk factors in later life periods, including adult body mass index (BMI) and adult SES (β = −8.2 cm^2^, 95% confidence interval [CI]: −13.3, −3.2). Highest vs. lowest parental educational attainment was associated with higher percent density in models that adjusted for age at mammogram and adult BMI (e.g., β = 4.8, 95% CI = 0.6, 9.1 for maternal education of college or higher degree vs. less than high school), but the association was attenuated and no longer statistically significant after further adjustment for early life factors. There were no associations between early life SES indicators and non-dense area after adjustment for adult BMI. Neither adult education nor adult income was statistically significantly associated with any measure of mammographic density after adjusting for age and adult BMI.

**Conclusions:**

We did not observe consistent associations between different measures of early life SES and mammographic density in adulthood.

## Background

Breast cancer risk is influenced by exposures that affect breast development and tissue structure and occur throughout the life-course, beginning with in utero and childhood periods [1]. The majority of research on early life determinants of breast cancer risk has focused on proxies for pre- and postnatal hormonal exposures including birthweight, maternal pre-eclampsia, twin membership and childhood growth [2–5]. Many of these exposures are in turn largely shaped by early life social environment, including parental socioeconomic status (SES) [6–10]. In addition to its relationship with early life hormonal exposures, early life social environment may represent other as yet unknown early life exposures that directly influence breast cancer risk. It can also indirectly influence breast cancer risk through shaping the development of risk factors for breast cancer operating in adolescence and adulthood, including reproductive events, lifestyle factors and adult socioeconomic circumstances.

Higher adult SES, particularly greater educational attainment, has consistently been associated with increased breast cancer risk [11–13], but only a few studies have focused on investigating the associations between early life socioeconomic factors and risk of breast cancer. A study of female participants in the Wisconsin Longitudinal Study reported a higher risk of breast cancer incidence in women with more educated mothers and higher childhood family income, and only partial mediation of these associations through adult risk factors for breast cancer, including adult SES [14]. A large Dutch study that considered multiple cancer sites including breast cancer reported no associations between childhood SES (as measured by paternal occupational class) and breast cancer risk, but found adult education to be positively associated with breast cancer risk [15]. These studies have relied on parental SES data reported by adolescent or adult offspring, and have lacked data on other perinatal and childhood factors. Although certain indicators of early life SES (e.g., parental education) may be easily recalled after an extended period of time, other SES indicators (e.g., family income) as well as other early life factors (e.g., birthweight) that may account for some of the association of breast cancer risk with early socioeconomic environment, may be reported with more difficulty and error [16–18]. Long-term prospective studies offer the best study design for examining early life influences on breast cancer risk. However, given the low incidence of breast cancer in the general population, the study population must be very large to allow for sufficient number of breast cancer cases, which presents many logistical and financial difficulties. As a feasible alternative, strong biomarkers of risk may be used in lieu of breast cancer incidence.

Using prospectively collected early life data, we examined the associations between early life SES (as defined by parental education and family income around the time of birth) and adult mammographic breast density, a measure of the amount of dense (fibroglandular) breast tissue as visualized on a mammogram and a strong and independent risk factor for future breast cancer risk [19–23]. To further elucidate the nature of the association between early life SES and mammographic density, we used several measures that capture different components of breast density and may suggest different pathways to breast cancer risk. These include the areas of the breast corresponding to the dense and nondense fatty tissue (dense and nondense areas, respectively), and the proportion of dense tissue area relative to total breast area (percent density). Dense area and percent density have similar positive associations with breast cancer risk and the majority of risk factors for breast cancer while nondense area is inversely associated with breast cancer risk [24–27]. We investigated the extent to which any observed associations were explained by 1) maternal and birth characteristics that have previously been associated with breast cancer risk, and 2) established risk factors for breast cancer in later life periods, including reproductive and lifestyle factors and adult SES. Persistent effects of the early socioeconomic environment on breast cancer risk after consideration of established risk factors across the lifecourse would suggest different directions from the current paradigm of breast cancer risk that chiefly addresses specific hormonal and growth related factors across the lifecourse.

## Methods

### Study sample and data collection

We used data from the Early Determinants of Mammographic Density (EDMD) Study, an adult follow-up study of participants from several U.S. birth cohorts, established to investigate early life exposures and breast cancer risk [28, 29]. The birth cohorts, the Child Health and Development Studies (CHDS) and the New England sites of the Collaborative Perinatal Project (CPP), were similar in design, recruitment and data collection protocols, and have been previously described in detail [30–33]. Briefly, pregnant mothers of participants were enrolled in 1959–1966 during their prenatal care visits and were followed through the prenatal period, labor and delivery, and their offspring (EDMD adult follow-up study participants) were followed from birth through early childhood. The CHDS enrolled pregnant women who were members of the Kaiser Foundation Health Plan and lived in the Oakland, California area. The CPP, a multi-center national pregnancy and birth cohort study, included two New England sites that enrolled pregnant women who lived near Boston, Massachusetts and Providence, Rhode Island and received prenatal care at the teaching hospitals affiliated with Harvard University and Brown University. As part of the EDMD adult follow-up study in 2005–2008, we traced 1925 female offspring in the CHDS and New England CPP and successfully enrolled 1134 women (59%) who represented a random sample meeting all of the following criteria: 1) were singleton birth, 2) had recorded weight and height at birth, 3) had recorded weight and height measures for two or more childhood time points, 4) participated in the last childhood study follow-up, and 5) had third trimester serum available and 6) had a sister enrolled in the original cohort. Our tracing rates were higher for the CHDS (80%) than for the CPP (59%), but the participation rates among women who were traced were similar for the CHDS (85%) and the CPP (88%). We restricted the current analysis to 893 women (79% of participants enrolled) who had no prior diagnosis of breast cancer, reported past or planned future mammograms and provided a signed medical release form for the study team to obtain their mammograms. We could not obtain mammograms for 23 participants, and excluded mammograms for 51 and 119 participants due to, respectively, poor quality and digital format of mammograms. The final sample of 700 participants consisted of 441 individuals and 127 sibling sets (122 sibling pairs and 5 sets of three siblings). All participants were born between 1959 and 1967, and were on average 43.1 years old (Standard deviation [SD] = 2.3, Interquartile range [IQR]: 41.7–44.6) at the time of their mammogram. Data on all early life factors were prospectively collected from mothers and their offspring during the prenatal period or around the time of birth and childhood visits. Data on factors in later life periods were collected through computer-assisted interviews with the participants in adulthood.

The study was approved by the Institutional Review Boards at Columbia University Medical Center, Kaiser Permanente, Brigham and Women’s Hospital and Brown University. All participants provided informed consent prior to data collection.

### Measures

#### Early life factors

Early life SES indicators included maternal and paternal education and annual family income around the time of each participant’s birth. Highest maternal and paternal educational attainment were categorized into less than high school graduate, high school graduate, trade or technical training or some college including associate degree, and college or higher degrees. We categorized childhood annual family income collected in US dollars into 5, 4 and 3 levels. The analysis using these different categorization of family income yielded the same overall results and we restrict our presentation to the categories of < $5000, $5000-7999 and ≥ $8000. We also considered additional early life data that have been associated with mammographic density [28, 34]. These included prenatal smoke exposure ([PTS] based on mother’s smoking status during pregnancy), mother’s birthplace (US vs. foreign), maternal age at pregnancy and participant’s birth order (first birth, second birth, third birth and fourth or fourth plus birth), all collected through interviews with pregnant mothers. Data on participants’ weights at birth and at age 4 years were also obtained from clinical records of these measures at birth and follow-up child visits (all in kg). We limit the presentation of our analysis to models including PTS, birthweight and weight at age 4 as these factors had the strongest associations with mammographic density in our study population.

#### Reproductive factors

We collected detailed reproductive history data from participants in adulthood and derived the following variables: age at menarche (in intervals of 0.5 years), age at first live birth (in years), parity (nulliparous and parous with number of live births), and menopausal status (premenopausal, perimenopausal and postmenopausal).

#### Adult lifestyle factors

We used data on current smoking status (never, former and current smokers), alcohol intake in last 12 months (nondrinker, <3 drinks/week, 3–7 drinks/week, >7 drinks/week), moderate to vigorous physical activity in the last 3 months (categorized into no exercise and tertiles of exercise in number of minutes per week) and current body mass index (BMI in kg/m^2^, calculated from self-reported height and weight).

#### Adult socioeconomic factors

Participants’ highest educational level and current family income were used as indicators of adult SES. Adult education was categorized into high school graduate or less, trade school or some college, bachelor degree, and master or higher degree. Adult income was categorized as follows: <$50,000, $50,000-75,000, $75,000-100,000 and > $100,000.

#### Mammographic density

We used cranio-caudal film mammograms taken closest to the date of interview, and used films from the right breast only if the left breast films were not available. To assess mammographic density, a trained investigator used a computer program (Cumulus) to outline the total breast area and dense tissue area. We converted the number of pixels within the outlined boundaries to cm^2^ in order to calculate the size of the areas corresponding to total breast and dense breast tissue. We then calculated percent mammographic density (hereafter percent density) by dividing the dense area by total breast area and multiplying the result by 100, and non-dense area by subtracting dense area from total breast area. We read mammograms in batches of about 50 films, with each batch including films from both cohort sites. All films for a participant and films from sibling sets were read together in the same batch. We re-read 10% of the films from each batch to measure within-batch reproducibility of readings. We also repeated reading on additional 10% of films from every batch to measure batch-to-batch variability. The within-batch correlation coefficient was 0.96 for percent density and the intra-class coefficient for between-batch reliability was 0.95.

### Statistical Analysis

We tested whether birth cohort site modified the associations between the three early life SES indicators (maternal and paternal education, family income) and three measures of density (percent density, dense area and nondense area) by including an interaction term between the cohort site and each early life SES indicator in the regression models of mammographic density. The interaction terms with early life SES and site were not statistically significant and the inclusion of the interaction term did not significantly improve the model fit. We therefore combined the data across the two cohort sites. As the results for paternal and maternal education at birth were similar in all analyses, we limit the presentation of our results to maternal education only.

We examined the association of each early life SES indicator and each mammographic density measure separately, using generalized estimating equation models for multivariable analysis to account for possible correlated data for sibling sets within the study sample. We began by fitting a minimally adjusted model that included age at mammogram and adult BMI. We added to this model other early life factors, followed by the addition of adult SES indicators of income and education. Finally, we added two sets of breast cancer risk factors at later life periods: 1) reproductive factors: age at menarche, age at first birth, menopausal status, and 2) lifestyle factors: smoking status, current alcohol consumption and physical activity. We repeated our final analyses using a combined parental education variable from paternal and maternal education, and stratified our multivariable models separately by race/ethnicity and menopausal status; however, the results of these additional analyses were not different from the overall results, and are therefore not presented.

## Results

Table 1 displays the distribution of key variables and breast cancer risk factors across the life-course. Participants were predominantly premenopausal (~70%) and of non-Hispanic white ethnic background (78%), and majority did not have a family history of breast cancer (89%). The means of mammographic density measures were as follows: 31.8% for percent density (IQR: 15.9–79.1), 35.8 cm^2^ for dense area (IQR: 20.5–47.7) and 102.1 cm^2^ for nondense area (IQR: 46.4–447.7). The distributions of paternal and maternal education were similar with over two-thirds of the participants having parents with high school or less education. Participants had relatively high educational attainment with over 80% obtaining some education beyond high school. Maternal smoking at the time of pregnancy with the study participants was relatively common (40%), while over half of the participants themselves never smoked regularly. Participants had low alcohol intake (36% did not consume alcoholic beverages in the last 12 months) and low physical activity levels (31% did not engage in any physical exercise in the last 3 months).

**Table 1:** Distribution of risk factors for breast cancer (*n* = 700), Early Determinants of Mammographic Density (EDMD) Study

	N (%) or mean ± SD
Age at mammogram (years)	43.1 ± 2.3
Race/ethnicity	
Non-hispanic white	546 (78.2)
African-American	86 (12.3)
Hispanic	40 (5.7)
Asian/pacific-islander/other	26 (3.7)
Maternal education	
Less than high school	180 (25.9)
High school graduate	289 (41.6)
Trade school or some college	150 (21.6)
College or higher education	75 (10.8)
Paternal education	
Less than high school	176 (27.4)
High school graduate	246 (38.3)
Trade school or some college	120 (18.7)
College or higher education	101 (15.7)
Childhood family income ($)	
< 5000	228 (35.4)
5000- < 8000	292 (45.3)
≥ 8000	125 (19.4)
Prenatal tobacco smoke (PTS) exposure to maternal Smoking	
No	406 (59.9)
Yes	272 (40.1)
Birthweight (kg)	3.4 ± 0.5
Weight at 4 years (kg)	16.8 ± 2.4
Age at menarche (years)	
< 12	135 (19.4)
12	192 (27.6)
13	200 (28.7)
≥ 14	169 (24.3)
Body mass index (kg/m^2^)	27.5 ± 6.4
Menopausal status	
Premenopausal	480 (69.7)
Menopausal transition	120 (17.4)
Postmenopausal	89 (12.9)
Adult education	
High school or less	130 (18.6)
Trade school or some college	259 (37.0)
Bachelor’s degree	214 (30.6)
Master’s degree or higher	97 (13.9)
Adult household income	
< 50,000	162 (23.9)
50,000- < 75,000	128 (18.9)
75,000- < 100,000	140 (20.7)
≥ 100,000	247 (36.5)
Adult smoking status	
Never smoker	377 (53.9)
Former smoker	219 (31.3)
Current smoker	103 (14.7)
Recent alcohol intake (drinks/week)	
Non-drinker	251 (36.0)
< 3	210 (30.1)
3–7	147 (21.1)
> 7	89 (12.8)
Recent physical exercise (minutes/week)	
No exercise	214 (30.6)
< 100	170 (24.3)
100–195	162 (23.2)
≥ 195	153 (21.9)
Mammographic density	
Percent breast density (%)	31.8 (18.7)
Dense breast area (cm^2^)	35.8 (22.0)
Non-dense breast area (cm^2^)	102.1 (75.0)

Table 2 presents the results of multivariable regression models separately for maternal education and family income at birth and each mammographic density measure, i.e., dense area (Panel 1), non-dense area (Panel 2) and percent density (Panel 3). Models 1 through 3 in each panel show the estimates of the associations between early life SES and density measures from the minimally adjusted model with age at mammogram and adult BMI, (Model 1), an early life model that further adjusts for PTS, weights at birth and age 4 years (Model 2), and a more fully adjusted model that include all previously included covariates as well as adult SES (Model 3). The highest relative to the lowest childhood family income category was associated with smaller dense area in all the models, including the final multivariable model that adjusted for adult BMI and adult SES (β = −8.2 cm^2^, 95% confidence interval [CI] = −13.3, −3.2, Panel 1, Model 3). Maternal education was not associated with dense area in any models. As compared with maternal education of less than a high school education, maternal educational attainment of college or higher degree was associated with higher percent density after adjustment for age at mammogram and adult BMI (β = 4.8, 95% CI = 0.6, 9.1; Panel 3, Model 1), but this association was reduced with adjustment for early life and adult factors in the fully adjusted model (β = 1.1, 95% CI = −4.0, 6.2; Panel 3, Model 3). There were no significant differences in percent density for other maternal education categories, or in dense area for other categories of childhood family income. Non-dense area was also not associated with either parental education and childhood family income in any of the models.

**Table 2:** Linear regression models of associations between early life socioeconomic status and adult mammographic density, EDMD Study

	Model 1: Adjusted for age at mammogram & adult BMI	Model 2: adjusted, for age at mammogram, adult BMI and early life factors (PTS, birthweight, weight at age 4)	Model 3:adjusted for age at mammogram, adult BMI, early life factors and adult SES (adult education & income)
	β (95% CI)	β (95% CI)	β (95% CI)
Dense area (cm^2^)			
Maternal Education at birth			
Less than high school (reference)			
High school graduate	0.01 (−4.3, 4.3)	−0.6 (−5.2, 3.9)	−1.7 (−6.3, 3.0)
Trade school or some college	−0.8 (−5.7, 4.2)	−2.9 (−8.0, 2.2)	−4.3 (−9.8, 1.1)
College graduate or higher education	1.8 (−4.4, 8.0)	−1.4 (−8.3, 5.4)	−4.8 (−12.1, 2.4)
Family income at birth			
< $5000 (reference)			
$5000-$7999	−1.2 (−5.1, 2.7)	−1.4 (−5.4, 2.6)	−1.5 (−5.7, 2.7)
≥ $8000	−6.3 (−11.0, −1.6)	−6.9 (−11.8, −2.1)	−8.2 (−13.3, −3.2)
Non-dense area (cm^2^)			
Maternal education at birth			
Less than high school (reference)			
High school graduate	−7.7 (−18.0, 2.6)	−5.4 (−15.6, 4.8)	−5.7 (−16.5, 5.0)
Trade school or some college	−1.0 (−13.4, 11.4)	−3.3 (−16.1, 9.6)	−3.8 (−17.0, 9.4)
College graduate or higher education	−8.2 (−20.8, 4.4)	−5.5 (−19.2, 8.3)	−3.5 (−18.6, 11.5)
Family income at birth			
< $5000 (reference)			
$5000-$7999	−4.3 (−13.2, 4.6)	−3.6 (−12.4, 5.2)	−1.9 (−11.1, 7.3)
≥ $8000	−1.9 (−13.2, 9.4)	−1.6 (−13.5, 10.2)	0.8 (−11.4, 12.9)
Percent density			
Maternal education at birth			
Less than high school (reference)			
High school graduate	1.4 (−1.4, 4.2)	0.7 (−2.2, 3.7)	0.4 (−2.6, 3.4)
Trade school or some college	0.5 (−2.8, 3.8)	−0.2 (−3.7, 3.3)	−0.8 (−4.4, 2.9)
College graduate or higher education	4.8 (0.6, 9.1)	3.4 (−1.4, 8.2)	1.1 (−4.0, 6.2)
Family income at birth			
< $5000 (reference)			
$5000-$7999	−0.7 (−3.2, 1.8)	−0.7 (−3.3, 1.8)	−0.9 (−3.6, 1.8)
≥ $8000	−2.6 (−6.1, 0.9)	−2.3 (−6.0, 1.4)	−3.2 (−6.9, 0.5)

*Abbreviations*: *PTS* prenatal tobacco exposure to maternal smoking during pregnancy, *BMI* Body mass index, *CI* confidence interval

Further adjustment for reproductive and lifestyle factors had minimal influences on the estimates of the associations for early life SES and either measure of density (data not shown).

Table 3 summarizes the associations separately for the two indicators of adult SES and dense area (Panel 1), non-dense area (Panel 2) and percent density (Panel 3). Adult education and income had similar positive associations with percent density and inverse associations with non-dense area in age-adjusted models (data not shown), but adjustment for adult BMI attenuated these associations considerably and no statistically significant associations remained. We observed no associations between adult SES and dense area in any of the models.

**Table 3:** Linear regression models of associations between adult socioeconomic status and adult mammographic density, EDMD Study

	Adjusted for age at mammogram and adult BMI
	β (95% CI)
Dense Area (cm^2^)	
Adult education	
High school or less	Ref.
Trade school or some college	0.8 (−3.8, 5.4)
Bachelor’s degree	2.5 (−2.3, 7.3)
Master’s degree or higher	3.8 (−2.2, 9.8)
Adult income	
< $50,000	Ref.
$50,000- < $75,000	−0.3 (−5.4, 4.8)
$75,000- < $100,000	0.3 (−4.9, 5.5)
≥ $100,000	0.6 (−4.0, 5.1)
Non-dense area (cm^2^)	
Adult education	
High School or Less	Ref.
Trade School or Some College	1.6 (−9.7, 12.9)
Bachelor’s Degree	−3.3 (−16.7, 8.06)
Master’s Degree or Higher	−0.3 (−13.5, 12.9)
Adult income	
< $50,000	Ref.
$50,000- < $75,000	−8.2 (−20.4, 3.9)
$75,000- < $100,000	0.5 (−12.3, 13.3)
≥ $100,000	0.2 (−11.2, 11.7)
Percent density	
Adult education	
High school or less	Ref.
Trade school or some college	0.1 (−3.2, 3.4)
Bachelor’s degree	2.0 (−1.6, 5.4)
Master’s degree or higher	3.1 (−0.9, 7.0)
Adult income	
< $50,000	Ref.
$50,000- < $75,000	0.01 (−3.6, 3.6)
$75,000- < $100,000	−0.8 (−4.4, 2.8)
≥ $100,000	−0.9 (−4.1, 2.3)

*Abbreviations*: *BMI* body mass index

## Discussion

In examining early life SES in relation to mammographic density, we aimed to further clarify the role of early life factors in the development of breast cancer risk, and identify possible life-course pathways. Specifically, we examined whether parental education and family income at birth were associated with mammographic density, assessed from mammograms obtained in midlife, after accounting for: 1) birth and childhood characteristics relevant to breast cancer risk, and 2) established risk factors for breast cancer in later life periods. We observed significantly smaller dense area for the highest family income level at birth and higher percent density for the highest parental educational attainment, but only the association between family income and dense area remained after adjustment for factors from different periods of life.

Percent density and dense area respectively capture the relative and absolute amount of fibroglandular tissues on mammograms. Both measures have similar positive associations with the risk of breast cancer and most risk factors for breast cancer [24, 27, 35]. Nondense area has been inversely associated with breast cancer risk although this association may not be independent of the effect of measures of dense area on risk [24]. Measures of body size such as BMI are more strongly associated with percent density and with nondense breast area than with dense area, as the former two measures reflect the amount of fat tissue in the breast [36]. In our study, adult BMI explained a large amount of variation in percent density and nondense area, including differences in these measures of density by parental education, suggesting that any influence from early life SES on relative amount of dense tissue may be working through influencing general body size and fat tissue in the breast, rather than directly influencing the amount of dense breast tissue, where breast tumors develop. This is further substantiated by the lack of any associations between parental education and dense area.

The patterns of parental education in percent density parallel well-documented positive associations between adult SES and breast cancer risk [11–13], which have also been reported in some studies of adult SES and mammographic density [26, 37]. However, we observed an opposite pattern for early life family income and dense area. Importantly, the inverse association between family income and dense area persisted and was minimally altered by accounting for breast cancer risk factors across the life course. Childhood family has been positively associated with breast cancer risk in limited research [14], but little is known about its association with mammographic density. Given that differences in dense area were only observed for the two extreme categories of early life family income and the unexpected direction of this association, our results should be interpreted with caution. In a recent study of the New York City site of CPP birth cohort, we reported a significant positive association between a composite measure of parental SES at birth, composed of parental education, income and occupation and percent density, but did not observe any associations for dense and nondense areas after accounting for early life factors [38]. Here, we examined the same composite early life SES measure available for the New England CPP, but not the CHDS, and did not find any associations with any measures of mammographic density. These CPP cohorts differ in their racial and ethnic diversity as well as overall sample size. Specifically, racial/ethnic minorities represented less than a quarter of the study population for the current analysis, but included over 60% of the New York-CPP cohort. Income and education are the most widely used indicators of SES in the U.S., and while these measures tend to be correlated, at times they show differential associations with health outcomes. In particular, these measures of SES are less strongly correlated, and individually may have weaker associations with many health outcomes in racial/ethnic minority populations [39, 40]. Nonetheless, our results, if replicated in other studies, point to the possibility that the associations between early life SES and mammographic density may be independent of and similar in strength as the associations with other established risk factors. Further research is also needed o clarify the direction of these associations, which is important for understanding whether SES influences on breast cancer risk is mediated through mammographic density.

The main strengths of our study include the collection of prospective data on early life factors and detailed data on breast cancer risk factors in adulthood, which together with highly reliable measures of mammographic density offer a thorough evaluation of the influence of early life socioeconomic factors on breast cancer risk. We lacked data for exposures during puberty and adolescence, critical life periods for breast cancer risk development, that are characterized by rapid growth, breast development and hormone production and slow differentiation of breast tissue [41–43]. Certain exposures such as BMI, cigarette smoking, and alcohol consumption in adolescence have been linked to breast cancer as well as mammographic density in a few studies [44–49], but focused research on SES during adolescence and subsequent breast cancer risk is extremely limited and merits further attention. Studies of mammographic density that rely on routine clinical mammography inherently include populations with greater access to healthcare. However, mammography is among the most widely used screening tests [50, 51]; nearly 80% of the participants in our adult follow up study population had undergone mammography, and thus, were included in this analysis. Participants with and without mammograms did not have statistically significant differences in many key factors in this analysis, including childhood family income, maternal and paternal education, maternal or self-reported race/ethnicity, birthweight and childhood weight, maternal smoking during pregnancy, age at follow up, BMI, menopausal status, adult SES and alcohol intake; the two groups were different in terms of adult smoking. Our study attained high tracing and participation rates, which were similar to or higher than other follow-up studies of other sites of the Collaborative Perinatal Project [52, 53]. Our study results are most generalizable to premenopausal and non-Hispanic white women, who comprised the majority of our study population. Mammographic density changes around the time of menopause, and different cross-sectional associations may be observed according to menopausal status [27, 54]. Racial/ethnic differences in mammographic breast density and/or its associations with breast cancer risk factors have been noted in some studies [55–59]. Studies examining life course SES and mammographic density in racially/ethnically diverse women are further warranted given the potential interaction between race/ethnicity and SES; however, currently few birth or prospective cohort studies with reliable early life data have sufficiently large representation of racial/ethnic minorities.

## Conclusions

In conclusion, we did not find consistent associations between early life SES and mammographic density in adulthood. The observed associations differed by the type of SES indicator and measure of mammographic density, and were limited to comparisons between the highest and lowest categories of early life SES. Higher parental education predicted higher percent density whereas higher parental family income predicted lower dense breast area, with the direction of the latter association being inconsistent with the known link between higher adult SES and increased breast cancer risk.

## Abbreviations

BMI: Body mass index
CI: Confidence interval
PTS: Prenatal tobacco smoke exposure to maternal smoking

## References

[1] Dos Santos Silva I, De Stavola BL, Kuh D, Hardy R (2002). Breast cancer aetiology: where do we go from here?. A life course approach to women’s health.

[2] Xue F, Michels KB (2007). Intrauterine factors and risk of breast cancer: a systematic review and meta-analysis of current evidence. Lancet Oncol.

[3] dos Santos Silva I, De Stavola BL, Hardy RJ, Kuh DJ, McCormack VA, Wadsworth ME (2004). Is the association of birth weight with premenopausal breast cancer risk mediated through childhood growth?. Br J Cancer.

[4] De Stavola BL, dos Santos Silva I, McCormack V, Hardy RJ, Kuh DJ, Wadsworth ME (2004). Childhood growth and breast cancer. Am J Epidemiol.

[5] Ahlgren M, Melbye M, Wohlfahrt J, Sorensen TI (2004). Growth patterns and the risk of breast cancer in women. N Engl J Med.

[6] Mortensen LH, Helweg-Larsen K, Andersen AM (2011). Socioeconomic differences in perinatal health and disease. Scand J Public Health.

[7] Astone NM, Misra D, Lynch C (2007). The effect of maternal socio-economic status throughout the lifespan on infant birthweight. Paediatr Perinat Epidemiol.

[8] Jansen PW, Tiemeier H, Looman CW, Jaddoe VW, Hofman A, Moll HA, Steegers EA, Verhulst FC, Mackenbach JP, Raat H (2009). Explaining educational inequalities in birthweight: the Generation R Study. Paediatr Perinat Epidemiol.

[9] Silva LM, Coolman M, Steegers EA, Jaddoe VW, Moll HA, Hofman A, Mackenbach JP, Raat H (2008). Low socioeconomic status is a risk factor for preeclampsia: the Generation R Study. J Hypertens.

[10] Shenkin SD, Starr JM, Deary IJ (2004). Birth weight and cognitive ability in childhood: a systematic review. Psychol Bull.

[11] Faggiano F, Partanen T, Kogevinas M, Boffetta P (1997). Socioeconomic differences in cancer incidence and mortality. IARC Sci Publ.

[12] Heck KE, Pamuk ER (1997). Explaining the relation between education and postmenopausal breast cancer. Am J Epidemiol.

[13] Robert SA, Strombom I, Trentham-Dietz A, Hampton JM, McElroy JA, Newcomb PA, Remington PL (2004). Socioeconomic risk factors for breast cancer: distinguishing individual- and community-level effects. Epidemiology.

[14] Pudrovska T, Anikputa B (2011). The role of early-life socioeconomic status in breast cancer incidence and mortality: unraveling life course mechanisms. J Aging Health.

[15] de Kok IM, van Lenthe FJ, Avendano M, Louwman M, Coebergh JW, Mackenbach JP (2008). Childhood social class and cancer incidence: results of the globe study. Soc Sci Med.

[16] Tehranifar P, Liao Y, Flom JD, Terry MB (2009). Validity of self-reported birth weight by adult women: sociodemographic influences and implications for life-course studies. Am J Epidemiol.

[17] Batty GD, Lawlor DA, Macintyre S, Clark H, Leon DA (2005). Accuracy of adults’ recall of childhood social class: findings from the Aberdeen children of the 1950s study. J Epidemiol Community Health.

[18] Terry MB, Flom J, Tehranifar P, Susser E (2009). The role of birth cohorts in studies of adult health: the New York women’s birth cohort. Paediatr Perinat Epidemiol.

[19] Boyd NF, Guo H, Martin LJ, Sun L, Stone J, Fishell E, Jong RA, Hislop G, Chiarelli A, Minkin S (2007). Mammographic density and the risk and detection of breast cancer. N Engl J Med.

[20] Boyd NF, Lockwood GA, Byng JW, Little LE, Yaffe MJ, Tritchler DL (1998). The relationship of anthropometric measures to radiological features of the breast in premenopausal women. Br J Cancer.

[21] Byrne C, Schairer C, Wolfe J, Parekh N, Salane M, Brinton LA, Hoover R, Haile R (1995). Mammographic features and breast cancer risk: effects with time, age, and menopause status. J Natl Cancer Inst.

[22] Oza AM, Boyd NF (1993). Mammographic parenchymal patterns: a marker of breast cancer risk. Epidemiol Rev.

[23] McCormack VA, dos Santos SI (2006). Breast density and parenchymal patterns as markers of breast cancer risk: a meta-analysis. Cancer Epidemiol Biomarkers Prev.

[24] Pettersson A, Graff RE, Ursin G, Santos Silva ID, McCormack V, Baglietto L, Vachon C, Bakker MF, Giles GG, Chia KS et al. Mammographic density phenotypes and risk of breast cancer: a meta-analysis. J Natl Cancer Inst. 2014;106(5):1–11. 10.1093/jnci/dju078 PMC4568991 24816206

[25] Pettersson A, Hankinson SE, Willett WC, Lagiou P, Trichopoulos D, Tamimi RM (2011). Nondense mammographic area and risk of breast cancer. Breast Cancer Res.

[26] Vachon CM, Kuni CC, Anderson K, Anderson VE, Sellers TA (2000). Association of mammographically defined percent breast density with epidemiologic risk factors for breast cancer (United States). Cancer Causes Control.

[27] Boyd NF, Martin LJ, Yaffe MJ, Minkin S (2006). Mammographic density: a hormonally responsive risk factor for breast cancer. J Br Menopause Soc.

[28] Terry MB, Schaefer CA, Flom JD, Wei Y, Tehranifar P, Liao Y, Buka S, Michels KB (2011). Prenatal smoke exposure and mammographic density in mid-life. J Dev Origins Health Dis.

[29] Tawfik H, Kline J, Jacobson J, Tehranifar P, Protacio A, Flom JD, Cirillo P, Cohn BA, Terry MB. Life course exposure to smoke and early menopause and menopausal transition. Menopause. 2015; 22(10):1076–83. 10.1097/GME.0000000000000444 PMC4580481 25803667

[30] Broman S: The Collaborative Perinatal Project: An Overview. In: Handbook of Longitudinal Research, Vol I. Volume I, edn. Edited by Mednick SA, Harway M, Finello KM: New York: Praeger Publishers; 1984: 185–227.

[31] Van den Berg BJ, Mednick SA, Harway M, Finello KM (1984). The California child health and development studies. Hanbook of longitudinal studies.

[32] van den Berg BJ, Christianson RE, Oechsli FW (1988). The California Child Health and Development Studies of the School of Public Health, University of California at Berkeley. Paediatr Perinat Epidemiol.

[33] Susser E, Buka SL, Schaefer CA, Andrews H, Cirillo PM, Factor-Litvak P, Gillman M, Goldstein JM, Ivey Henry P, Lumey LH (2011). The early determinants of adult health study. J Dev Origins Adult Health Dis.

[34] Park SK, Kang D, McGlynn KA, Garcia-Closas M, Kim Y, Yoo KY, Brinton LA (2008). Intrauterine environments and breast cancer risk: meta-analysis and systematic review. Breast Cancer Res Treat.

[35] Vachon CM, van Gils CH, Sellers TA, Ghosh K, Pruthi S, Brandt KR, Pankratz VS (2007). Mammographic density, breast cancer risk and risk prediction. Breast Cancer Res Treat.

[36] Stone J, Warren RM, Pinney E, Warwick J, Cuzick J (2009). Determinants of percentage and area measures of mammographic density. Am J Epidemiol.

[37] Aitken Z, Walker K, Stegeman BH, Wark PA, Moss SM, McCormack VA, Silva Idos S: Mammographic density and markers of socioeconomic status: a cross-sectional study. BMC Cancer. 10:35;1–11. 10.1186/1471-2407-10-35 PMC2829497 20144221

[38] Akinyemiju TF, Tehranifar P, Flom JD, Liao Y, Wei Y, Terry MB (2016). Early life growth, socioeconomic status, and mammographic breast density in an urban US birth cohort. Ann Epidemiol.

[39] Braveman PA, Cubbin C, Egerter S, Chideya S, Marchi KS, Metzler M, Posner S (2005). Socioeconomic status in health research: one size does not fit all. JAMA.

[40] Braveman P, Cubbin C, Marchi K, Egerter S, Chavez G (2001). Measuring socioeconomic status/position in studies of racial/ethnic disparities: maternal and infant health. Public Health Rep.

[41] John EM, Terry MB, Keegan TH, Bradbury AR, Knight JA, Chung WK, Frost CJ, Lilge L, Patrick-Miller L, Schwartz LA et al. The LEGACY Girls Study: Growth and development in the context of breast cancer family history. Epidemiology. 2016. 10.1097/EDE.0000000000000456 PMC5341739 26829160

[42] Trichopoulos D, Adami HO, Ekbom A, Hsieh CC, Lagiou P (2008). Early life events and conditions and breast cancer risk: from epidemiology to etiology. Int J Cancer.

[43] Colditz GA, Frazier AL (1995). Models of breast cancer show that risk is set by events of early life: prevention efforts must shift focus. Cancer Epidemiol Biomarkers Prev.

[44] Baer HJ, Tworoger SS, Hankinson SE, Willett WC (2010). Body fatness at young ages and risk of breast cancer throughout life. Am J Epidemiol.

[45] Yochum L, Tamimi RM, Hankinson SE (2014). Birthweight, early life body size and adult mammographic density: a review of epidemiologic studies. Cancer Causes Control.

[46] Vachon CM, Sellers TA, Janney CA, Brandt KR, Carlson EE, Pankratz VS, Wu FF, Therneau TM, Cerhan JR (2005). Alcohol intake in adolescence and mammographic density. Int J Cancer.

[47] Flom JD, Ferris JS, Tehranifar P, Terry MB (2009). Alcohol intake over the life course and mammographic density. Breast Cancer Res Treat.

[48] Rosenberg L, Boggs DA, Bethea TN, Wise LA, Adams-Campbell LL, Palmer JR (2013). A prospective study of smoking and breast cancer risk among African-American women. Cancer Causes Control.

[49] Jacobsen KK, Lynge E, Vejborg I, Tjonneland A, von Euler-Chelpin M, Andersen ZJ (2016). Cigarette smoking and mammographic density in the Danish Diet, Cancer and Health cohort. Cancer Causes Control.

[50] Centers for Disease Control and Prevention (CDC) Cancer screening - United States, 2010. MMWR Morb Mortal Wkly Rep. 2012;61(3):41–45. 22278157

[51] American Cancer Society (2013). Breast Cancer Facts & Figures 2013–2014.

[52] Hemachandra AH, Howards PP, Furth SL, Klebanoff MA (2007). Birth weight, postnatal growth, and risk for high blood pressure at 7 years of age: results from the Collaborative Perinatal Project. Pediatrics.

[53] Klebanoff MA, Zemel BS, Buka S, Zierler S (1998). Long-term follow-up of participants in the Collaborative Perinatal Project: tracking the next generation. Paediatr Perinat Epidemiol.

[54] Boyd N, Martin L, Stone J, Little L, Minkin S, Yaffe M (2002). A longitudinal study of the effects of menopause on mammographic features. Cancer Epidemiol Biomarkers Prev.

[55] Tehranifar P, Reynolds D, Flom J, Fulton L, Liao Y, Kudadjie-Gyamfi E, Terry MB (2011). Reproductive and menstrual factors and mammographic density in African American, Caribbean, and white women. Cancer Causes Control.

[56] McCormack VA, Perry N, Vinnicombe SJ, Silva Idos S (2008). Ethnic variations in mammographic density: a British multiethnic longitudinal study. Am J Epidemiol.

[57] Chen Z, Wu AH, Gauderman WJ, Bernstein L, Ma H, Pike MC, Ursin G (2004). Does mammographic density reflect ethnic differences in breast cancer incidence rates?. Am J Epidemiol.

[58] Habel LA, Capra AM, Oestreicher N, Greendale GA, Cauley JA, Bromberger J, Crandall CJ, Gold EB, Modugno F, Salane M (2007). Mammographic density in a multiethnic cohort. Menopause.

[59] El-Bastawissi AY, White E, Mandelson MT, Taplin S (2001). Variation in mammographic breast density by race. Ann Epidemiol.

